# A Sublethal Concentration of Sulfoxaflor Has Minimal Impact on Buff-Tailed Bumblebee (*Bombus terrestris*) Locomotor Behaviour under Aversive Conditioning

**DOI:** 10.3390/toxics11030279

**Published:** 2023-03-18

**Authors:** Laura James, Andrew M. Reynolds, Ian R. Mellor, T. G. Emyr Davies

**Affiliations:** 1 Protecting Crops and the Environment, Rothamsted Research, West Common, Harpenden AL5 2JQ, UKandy.reynolds@rothamsted.ac.uk (A.M.R.); 2 Faculty of Medicine & Health Sciences, School of Life Sciences, University of Nottingham, Nottingham NG7 2RD, UK; ian.mellor@nottingham.ac.uk

**Keywords:** bee, pesticide, bumblebee, behaviour, learning, sulfoximine, neonicotinoid, aversive, power law

## Abstract

Pesticide exposure has been cited as a key threat to insect pollinators. Notably, a diverse range of potential sublethal effects have been reported in bee species, with a particular focus on effects due to exposure to neonicotinoid insecticides. Here, a purpose-built thermal–visual arena was used in a series of pilot experiments to assess the potential impact of approximate sublethal concentrations of the next generation sulfoximine insecticide sulfoxaflor (5 and 50 ppb) and the neonicotinoid insecticides thiacloprid (500 ppb) and thiamethoxam (10 ppb), on the walking trajectory, navigation and learning abilities of the buff-tailed bumblebee (*Bombus terrestris audax*) when subjected to an aversive conditioning task. The results suggest that only thiamethoxam prevents forager bees from improving in key training parameters (speed and distanced travelled) within the thermal visual arena. Power law analyses further revealed that a speed–curvature power law, previously reported as being present in the walking trajectories of bumblebees, is potentially disrupted under thiamethoxam (10 ppb) exposure, but not under sulfoxaflor or thiacloprid exposure. The pilot assay described provides a novel tool with which to identify subtle sublethal pesticide impacts, and their potential causes, on forager bees, that current ecotoxicological tests are not designed to assess.

## 1. Introduction

Although bees have long been used as a model species in which to study learning and memory, and there is a large body of research that has been carried out on choices made in the field, or free flight choices in the laboratory, most alternative studies have focused on the use of appetitive learning in the Western honeybee (*Apis mellifera*). Furthermore, a single Pavlovian learning protocol, using olfactory conditioning and the proboscis extension response (PER), has typically been employed. Aversive conditioning protocols (e.g., using quinine) have also been developed for the honeybee, most often utilizing the sting extension reflex (SER) [1]; however, there remains a dearth of aversive methodologies, particularly in respect to non-*Apis* bee species. To address this deficit, we recently developed and optimised a thermal–visual arena experimental platform for aversive (place-learning) conditioning of the buff-tailed bumblebee (*Bombus terrestris* audax). The laboratory assay was used to demonstrate the existence of underlying movement regularities within the trajectories of walking bumblebees, directed via the speed–curvature power law, whereby motor control dictates a constrained relation between the kinematic property of speed and the geometric property of curvature [2]. This novel aversive conditioning (heat avoidance) and movement analysis (adherence to the speed–curvature power law) assay is further utilised here to assess the potential impacts of three common Group 4 insecticides [3], sulfoxaflor, thiamethoxam and thiacloprid, on the locomotion, navigation and learning abilities of buff-tailed bumblebees.

The neonicotinoid insecticide thiamethoxam has been reported as having a range of sublethal effects at field realistic exposure levels on honeybees, displaying a reduced larval and pupal survival and decreased adult emergence and survival [4,5,6,7,8], impaired flight and decreased homing success [5,9], impaired locomotion, organ disruptions [10], immunosuppression [11], decreased motor function and hyper activity [9] and also on bumblebees, with impacts on worker survival, brood production, food consumption, odor learning and egg laying [12,13,14]. Conversely, the neonicotinoid thiacloprid is generally reported as ‘not harmful to bees’ and is an insecticide which “poses no risk to bees (when applied according to the label) and can be applied to flowering crops” [15]. Furthermore, differential, lower toxicity has been reported for thiacloprid in honeybees in comparison to thiamethoxam and other neonicotinoids [16]. Some sublethal effects linked to thiacloprid have however recently been reported in studies on honeybees, potentially affecting olfactory learning and memory [17], foraging behavior, immunosuppression [18], selected parameters of antioxidative defence [19], homing success, communication and navigation [20,21], and in bumblebees, affecting colony development and reproduction [22,23].

The sulfoximine insecticide, sulfoxaflor, has been proposed as a potential replacement for the neonicotinoid insecticides thiamethoxam, clothianidin and imidacloprid that are banned for outdoor use in the European Union. Sulfoximine insecticides have the same molecular target as the neonicotinoids, as an insect nicotinic acetylcholine receptor (nAChR) agonist. However, the sulfoximines have a distinct mode of action, with promising effects against neonicotinoid resistant sap-sucking pests including aphids, whiteflies, hoppers, and Lygus, and are potentially less harmful to non-target insects [24,25]. To date there are only a handful of studies considering the sublethal effects of sulfoxaflor, some of which suggest that there may be negative fitness impacts of exposure on bumblebee colonies, with worker production and reproductive output reduced [26,27,28], possible impacts on bee oxidative stress leading to early onset of apoptosis and mortality [29] and impact on pollen collection leading to a reduction in the size of workers [30,31]. Nevertheless, other studies have reported no negative effects on olfactory conditioning or working memory [27] and no direct effect on larval mortality except when applied in combination with the fungal parasite *Noesma bombi* [32]. Studies in honeybees suggest that sublethal levels of sulfoxaflor can result in wing deformation in newly emergent bees [33], negatively affect foraging activity [34] and homing ability [35]. The existing research is therefore by no means extensive, or conclusive, and has largely looked at acute exposure regimes. Sulfoxaflor’s similarity to the neonicotinoids, in both its insect target (nAChRs) and systemic mode of action, does warrant its further study in relation to its potential to have detrimental effects on bee learning and memory.

As honeybees and bumblebees can regularly forage over large distances, their ability to relocate the hive is key to their survival. In honeybees, it has been shown that such navigational proficiencies rely heavily on a ‘celestial compass’, governed by polarised light [36] and using optic flow as an odometer to determine the distance travelled from the hive [37]. However, bees also utilise visual landmarks to navigate to and from the hive [38,39,40,41,42], using orientation flights to develop a visual memory of landmarks and goal location [43]. Therefore, when a forager honeybee re-encounters these landmarks, they inform which action should be performed next, for example, to approach the landmark [36], turn left or right [44], or in a particular compass direction [45]. In this way, visual and spatial cues can be used to teach bees novel learning tasks [40,46,47,48]. Recently it has been demonstrated that bumblebees will strategically use ground level linear features (such as roads) in their navigation [49].

Within bee colonies, detection of temperature fluxes and tight thermoregulation are vital to the health of the colony. Hive temperature is critical to honeybee brood development and changes in brood temperature of even 0.5 °C have been shown to have a significant influence on bee health, mortality, behavioural and morphological defects [50,51,52]. If hive temperature becomes too high then bees can implement behavioural mechanisms, such as fanning, to remove the hot air or collect water to cool the hive back to the optimum temperature. When the hive temperature drops too low, bees can generate metabolic heat through flight muscle vibrations or, as bumblebees do, build a waxy covering over the brood to keep the heat in. These behavioural modifications under temperature stress suggest that bees have highly adept temperature detection systems, allowing these behavioural modifications to be implemented. Additionally, an ability to detect and respond to temperature is not just applicable to avoidance of over or under heating by bees, as bumblebees, honeybees and solitary bees have been shown to be adept at detecting flowers based on floral differences in temperature patterns, utilising thermal detectors in their tarsi and antenna [53,54,55,56,57]. Some floral heat patterns are as much as 11 °C warmer than the surrounding flower [55]. More importantly, bumblebees have been shown to be able to use these heat patterns to discern which flowers have the highest floral rewards [55], and it is likely that these heat signals work in conjunction with other floral cues (e.g., scent and colour) to attract pollinators to floral nectar rewards. This suggests that temperature is an integral and reliable cue to bees, involved in both avoidance (of too high temperatures) and attraction (in terms of floral reward signalling), making it an apt environmental stimulus to utilise in bee conditioning. Specific thermal receptors for peripheral temperature detection have been identified in the honeybee, for example, the Hymenoptera specific Transient Receptor Potential Ankyrin (HsTRPA), which has been identified in many sensory structures, including the legs, proboscis and antennae [58,59]. Although bees clearly have the means to detect and perceive high temperatures as aversive stimuli, studies utilising temperature as an aversive stimulus are extremely rare. In the thermal–visual arena, we exploit bees’ exceptional spatial memory and thermal sensory abilities, over a series of training trials, to teach foragers to utilise visual cues to locate a (cool) reward zone. The arena uses an aversive (high temperature) conditioning paradigm, with elevated environmental temperature as a conditioning stimulus.

As movement data can be used to garner information about a wide array of species traits, such as behavior, interactions with individuals (conspecifics and other animals) and landscapes, migration and dispersal [60,61,62,63], animal movements are now being studied in novel and exciting ways in the emerging field of movement ecology [64]. Automated tracking of animal movements has made a large contribution to this field and reduced the need for continual, direct observation of subjects over long time periods [65,66]. A better understanding of animal movement patterns over a range of spatial scales can facilitate a better understanding of complex ecological systems and will only increase in importance in conservation strategies as human populations expand and new environmental stressors emerge [67]. Power laws (i.e., relationships in which a relative change in one quantity gives rise to a proportional relative change in the other quantity, independent of the initial size of those quantities) governing movement are said to be ‘scale-free’ i.e., both short and long values can occur, and no scale is more frequent (dominant) than another [68]. Increasingly, power law distributions have been found in the movement patterns of a wide range of animals [61,68,69,70,71], including *B. terrestris* [2]. The discovery of power laws (the speed–curvature power law) in the movement trajectories of bees facilitates the study of how a bees’ movement patterns may change in response to stressors such as pesticides. Being able to better understand basal behavioral templates behind bees’ locomotive trajectories may thereby provide a critical tool for the study of fine-scale sublethal pesticide effects, which is sorely lacking from current ecotoxicological frameworks (as highlighted by the new EFSA Draft bee guidance [72]). Biotic stressors, such as disease load, have been demonstrated to lead to deviations from optimal behavioral templates in primates, seabirds and humans [73,74,75]. Nonetheless, in honeybees, optimal Lévy flight characteristics [76] are not disrupted by infection with either *Nosema* sp. or Deformed Wing Virus (DWV) [77]. The robustness of the Lévy search patterns observed in honeybees may be due to Lévy flights being fundamental characteristics of neuronal processes, which are therefore unaffected by the physiological impacts of stressors such as disease. However, as we discussed extensively in James et al. [2], it is likely that the speed–curvature power law is governed by biomechanical constraints [78,79] and may therefore be responsive to physiological stressors. Further study of the speed–curvature power law in relation to bee movement patterns may therefore provide a key diagnostic tool to determine the underlying drivers of observed sublethal effects, allowing us to unpick potential causation and impact of biological stressors at a finer physiological scale. We predict that some pesticide exposure regimes may lead to non-optimal movements in *B. terrestris*, and this would be reflected in a change in the optimal power law relationships observed in James et al. [2].

In this pilot study, our results demonstrate that, of the three insecticidal compounds tested, only thiamethoxam prevented forager bees from improving in two of the key training parameters (speed and distanced travelled) within the thermal visual arena. Power law analyses revealed that the speed–curvature power law governing the walking trajectories of bumblebees is also potentially disrupted under thiamethoxam exposure. No equivalent effects were observed for either thiacloprid or the sulfoximine insecticide sulfoxaflor, suggesting that the training arena provides a novel tool for dissecting these finer-scale effects between compounds.

## 2. Materials and Methods

### 2.1. The Thermal–Visual Arena

**Figure 1:**
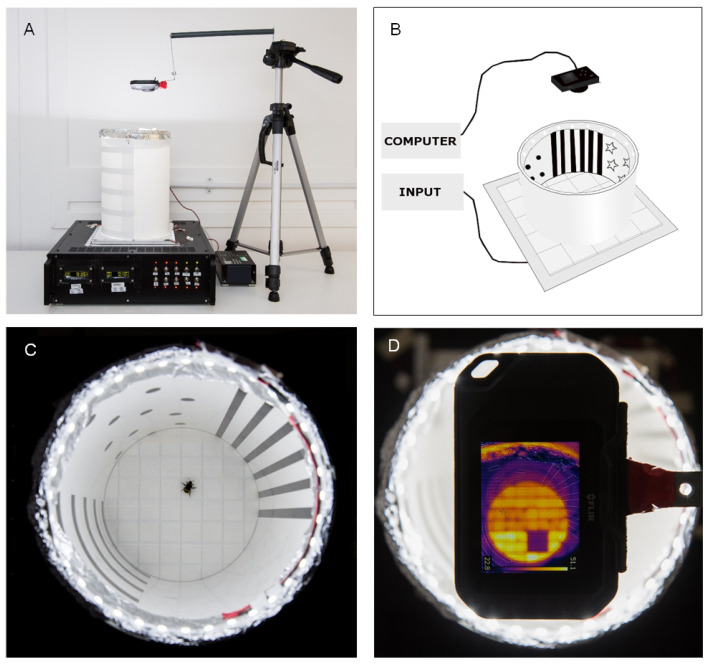
Taken from James et al. [2]; The thermal–visual arena. (**A**,**B**) Arena set up, with thermal imaging camera placed above the arena. (**C**) Birds-eye view of the arena displaying circumference and patterns. (**D**) Thermal image of the reward platform, showing the cool reward zone tiles (in purple).

The thermal–visual arena (Figure 1) is an aversive place learning assay for bumblebees [2]. The arena is based upon a design used by Ofstad, Zuker, and Reiser [80] to study walking Drosophila trajectories. To facilitate control of the arena’s temperature, the arena floor consists of a Peltier array of 64 (2.5 × 2.5 cm) individually controllable thermoelectric Peltier elements arranged in an 8 × 8 grid. The grid is covered in white masking tape to create a conspicuous, featureless surface which can be easily cleaned and replaced between trials to prevent scent marking by foragers. This surface also facilitates easy tracking of a dark bee silhouette on a light, white background (Figure 1C). A thermal imaging camera (FLIR C2 compact thermal camera, FLIR Systems UK, West Malling, Kent, UK) fixed above the arena (Figure 1A,D) allows for confirmation that no large-scale thermal gradients exist across the platform during trials, which may have influenced test subjects. A Perspex tube placed onto the Peltier platform creates the arena walls (Figure 1A). A ‘landscape’ of visual patterns is adhered to the surface of the tube’s circumference to create a visual landscape consisting of repeating patterns of horizontal bars, stars, dots, and vertical bars, denoting four quadrants of the circumference (Figure 1B,C). Light-emitting diodes (LEDs) (2100 lumens, colour temperature 6500 K) around the top edge of the arena (Figure 1C) are used to light the arena consistently above the bee flicker fusion frequency to prevent potential behavioural disturbances [81]. The arena is housed in a controlled environment room maintained at 22 °C on a day:night cycle of 16:8 h.

### 2.2. Bee Colonies

Two queen-right colonies of *B. terrestris* audax were obtained from Biobest (Biobest, Westerlo, Belgium); each colony contained a queen and approximately 200 workers. Bees were settled in wooden nest boxes (29 × 21 × 16 cm). Hives were provided with Biogluc (62% sugar concentration consisting of 37.5% fructose, 34.5% glucose, 25% sucrose, 2% maltose, and 1% oligosaccharides) (Biobest, Westerlo, Belgium) in two gravity feeders, modified from laboratory falcon tubes by puncturing small holes to allow feeding within a Perspex foraging tunnel connected to the hive. Hives were given a regular supply of pollen directly into the hive to allow ad libitum feeding.

### 2.3. Bee Training Protocol

The training protocol for all treatment groups was identical, with each bee given individual access to the thermal–visual arena for ten, three-minute, aversive training trials across three days. The aversive environment is created by heating the majority of the Peltier tiles of the thermal–visual arena’s floor to 45 °C and simultaneously cooling an area of four adjacent Peltier tiles to 25 °C to create a cool reward zone. As demonstrated in James et al. [2], the 45 °C arena floor provides strong aversive motivation for bumblebee foragers to locate and remain in the cool reward zone.

### 2.4. Age Cohorts and Marking

Bees were tagged with coloured bee marking discs (EH Thorne, Market Rasen, UK) within weekly age cohorts, and only bees of the same age cohort were used within each trial. Age cohorts were monitored to record active foragers who regularly left the hive to collect Biogluc from the feeders. Of the active foragers recorded in each age cohort, 12 were randomly selected to be used in each trial. All forager age cohorts were one-week post-emergence when used in trials to standardise forager age.

### 2.5. Choice of Pesticide Compounds and Exposure

*B. terrestris* foragers were exposed to one of three insecticide compounds to assess impacts on behavioural and movement parameters in the thermal–visual arena. As well as testing the sulfoximine compound sulfoxaflor, two neonicotinoid insecticides were also tested. The neonicotinoids thiamethoxam and thiacloprid were selected as although thiamethoxam is one of the three neonicotinoids now banned for outdoor use in the EU, it is still widely used in the rest of the world. Thiacloprid was selected as it is one of the cyano-substituted neonicotinoids generally considered to have lower bee toxicity and therefore is not part of the EU moratorium [16].

The 12 selected foragers were randomly allocated, three bees per treatment, to one of four treatments: (1) control (clean Biogluc—no insecticide dosage given), (2) thiacloprid, (3) thiamethoxam or (4) sulfoxaflor. Three replicates of each trial experiment were carried out to give a total of 9 data points (bees) per treatment. Foragers’ wings were clipped using a queen marking cage and dissection scissors (EH Thorne, Market Rasen, UK), to ensure bees walked on the test surface. Bees were then housed in individual Perspex cages with access to a modified 1.5 mL Eppendorf tube feeder to facilitate the assessment of individual food consumption. Bees were exposed to the insecticide compounds through feeding on dosed Biogluc sugar syrup (1 mL per individual feeder). All insecticides were technical grade and not formulation. Insecticide-exposed bees were compared against control bees which were given clean Biogluc solution with no insecticide. A sublethal concentration of each compound was provided in each feeder (sulfoxaflor 5 ppb, thiacloprid 500 ppb, thiamethoxam 10 ppb), equating to concentrations taken from literature reports of residue levels found in pollen and nectar in the field (see Section 2.6.2). For sulfoxaflor, a ‘higher’ sublethal concentration (designated as ten times the lower sublethal concentration; 50 ppb) was also included in our study. Due to the nature of the assay, bees are required to walk within the thermal–visual arena to locate the cool reward zone. Therefore, using sublethal insecticide dosages which would still maintain bee mobility was essential.

### 2.6. Trial Design

Three *B. terrestris* foragers were tested for each insecticide treatment (lower sublethal and higher concentration trials) and three replicates were conducted for each experiment, so that in total we tested 9 bees per insecticide treatment under lower (or higher) insecticide concentration conditions. All bees were one-week post emergence and of the same age cohort when used in the trials.

#### 2.6.1. Chronic Pesticide Exposure and Temporal Spacing of Trials

Bee trials were run sequentially from bee 1 to bee 12 for each set of trials, and treatments were randomised across bees to limit the influence of bee order. The order in which bees were run was also randomized prior to trial 1 and then remained the same throughout the trials (1–10) to ensure relatively equal temporal spacing between trials. Chronic pesticide exposure was conducted over a six-day period, three days prior to the start of training trials and then continuing for the three days of the trials. Temporal spacing of trials was consistent, with trials 1–4 conducted on one day, trials 5–7 on the next and trials 8–10 on the final day.

#### 2.6.2. Sublethal Concentration Insecticide Trials

A sublethal thiamethoxam concentration of 10 ppb was selected, based on studies reporting thiamethoxam residues found in the nectar and pollen of treated plants in the field [82,83,84,85,86,87] and previous studies which have also used this concentration as a field realistic exposure scenario [88,89,90]. A thiacloprid concentration of 500 ppb was used, based on field nectar residues reported by Ellis et al. [22]. Two concentrations of sulfoxaflor were tested: a lower concentration of 5 ppb, based on a predicted field-realistic concentration used by Siviter et al. [26,27,91,92] in the only sulfoxaflor bumblebee exposure studies that had been conducted when these trials were designed, and a higher concentration equating to 50 ppb. This later value represents an extreme exposure scenario, to examine the potential worst-case effects (although likely unrealistic to a field setting). These dosages were achieved through serial dilution of insecticides with acetone and water and then final dilutions into Biogluc. Bees were exposed to the appropriate test concentrations of thiacloprid, thiamethoxam and sulfoxaflor as detailed in Section 2.5. A schematic summary of the trials is available as Supplementary Figure S1.

A total of 12 bees were tested for the thiacloprid, thiamethoxam and sulfoxaflor lower (sublethal) concentration trials. Each experimental replicate represented a different age cohort from the same hive (Hive 1). Replicates 1–3 comprised 3 sulfoxaflor, 3 thiacloprid, 3 thiamethoxam and 3 control bees per trial (×3). Similarly, a total of 6 bees × 3 replicates = 18 bees were employed as controls or tested at the higher sulfoxaflor concentration specified above (9 bees tested per treatment in total). Each replicate represents a different age cohort from the same hive (Hive 2).

#### 2.6.3. Food Consumption Recording

On day 1 of the experiments, each bee was individually caged and given access to a single 1.5 mL Eppendorf feeder. Individual (empty) Eppendorf feeders were weighed, and their weight recorded. Feeders were filled with a standardised amount (1 mL) of Biogluc (±insecticide) solution and reweighed. Each subsequent day of the experiment (day 2–7), feeders were weighed to assess individual food consumption, emptied, cleaned, refilled, and reweighed. Control evaporation feeders were also set up in empty cages to determine potential food loss through evaporation and ensure accuracy of food consumption data. The average of the evaporation from the five evaporation tests per day was calculated as 0.022 g. This value was therefore taken away from all bee consumption values prior to data analysis, to take loss through evaporation into account.

#### 2.6.4. Trial Recording and Video Processing

All trials were recorded using a FLIR C2 thermal camera (FLIR Systems UK, West Malling, Kent, UK) situated above the arena. Debut Video Capture Software, Version 5 (NCH Software, Inc., 6120 Greenwood Plaza Blvd, Greenwood Village, CO, USA) was used to capture video recordings. Recorded video files were tracked with idTracker using custom parameters [93].

### 2.7. Data Analyses

Sample sizes for the trials were *n* = 12, 3 bees per treatment (control, thiacloprid, thiamethoxam, sulfoxaflor). Each trial was replicated 3 times to give a total of 9 data points per treatment.

#### 2.7.1. Training Parameters

Training parameter data for each bee were produced from tracking files using custom R scripts (Jess Evans, Statistics Department, Rothamsted). A range of training parameters were assessed for both trial 1 (pre-training) and trial 10 (post-training) for all bees in each treatment, including bee trajectory maps (the route the bee takes within the arena during the trial), time the bee spent in the reward zone, the distances bees travelled throughout the trial and the average speed of the bee throughout the trial. All statistical analyses of training parameters were conducted in GraphPad Prism 8.2.1 (GraphPad Software, 2020). A suite of normality tests (Anderson-Darling, D’Agostino and Pearson, Shapiro-Wilk and Kolmogorov-Smirnov) were run on all datasets. ANOVA tests with multiple comparisons were conducted with a Tukey’s correction for multiple comparisons.

#### 2.7.2. Power Law Calculation

Raw trajectory data outputted by idTracker were used for power law calculation. Power law exponents were calculated based on individual bee’s post-training trials (trial 10), to see the effect that insecticide exposure regimes had on the most trained phenotype (as bees will have learnt the task to the best of their ability by trial 10). This also allows the effect of training on the power law exponent to be examined and whether this changes over time.

To assess whether the exponent (β) of the speed–curvature power law relationship changed under different insecticide exposure regimes, exponents of bees in each treatment during the post training trial (trial 10) were calculated (Supplementary Table S1). Speed–curvature power law calculation was identical to the method used in James et al. [2]. For the data analysis, the *x*, *y* coordinates and corresponding timestamps for whole trajectories, for individual bees, from the centroid tracking were used w to compute angular speed *A*(*t*) and curvature *C*(*t*) using standard differential geometry. Velocities were calculated from consecutive, regularly timed, positional fixes,

(1) $$\dot{x}=\frac{x\left(t+\Delta t\right)-x\left(t\right)}{\Delta t}\;\mathrm{and}\;\dot{y}=\frac{y\left(t+\Delta t\right)-y\left(t\right)}{\Delta t}$$

where Δt=0.2 s is the time interval between consecutive recordings. Accelerations $\ddot{x}$ and $\ddot{y}$ were calculated in a directly analogous way from consecutive velocities. Together these quantities determine the radius of curvature,

(2) $$R=\left|\frac{{\left({\dot{x}}^{2}+{\dot{y}}^{2}\right)}^{3/2}}{\dot{x}\ddot{y}-\dot{y}\ddot{x}}\right|$$

which in turn gives the angular speed,

(3) $$A={\left({\dot{x}}^{2}+{\dot{y}}^{2}\right)}^{1/2}/R$$

and the curvature,

(4) C=1/R

All data passed normality testing (Shapiro-Wilk test, D’Agostino and Pearson test, *p* ≧ 0.05) and therefore parametric statistical tests were used to assess whether there were treatment differences in speed–curvature power law exponents (β). ANOVAs with multiple comparisons were used to compare power law exponents across treatment groups.

## 3. Results

### 3.1. How Does Chronic Insecticide Exposure Affect Food Consumption?

**Figure 2:**
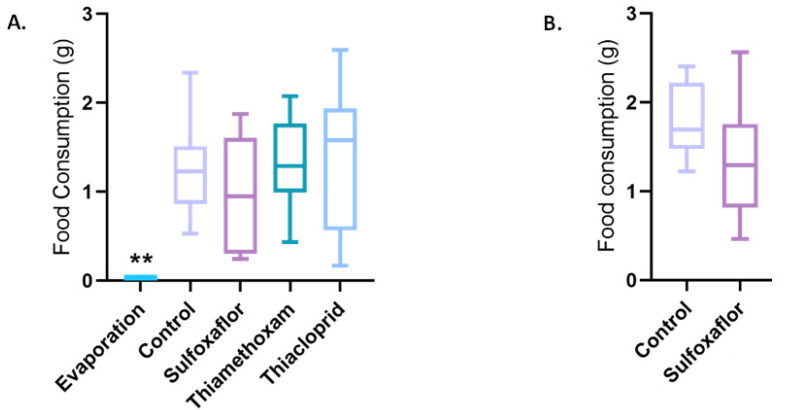
Food consumption across treatments. (**A**) Total 5-day food consumption of bees in the lower concentration trials compared to evaporation feeders. One-way ANOVA with Tukey correction for multiple comparisons, *p* values: evaporation vs. control (0.0049 **), evaporation vs. thiacloprid (0.0035 **), evaporation vs. sulfoxaflor (0.0275) and evaporation vs. thiamethoxam (0.0034 **). (**B**) Total 5-day food consumption of bees in the higher concentration trials.

Figure 2A shows the total amount of food consumed over the five days of the lower concentration trials by bees in each treatment (see also Supplementary Table S2A). All treatments consumed significantly more food than that lost in the evaporation test feeders, as would be expected. There were no other significant differences in the amount of food consumed between any of the other treatments in the lower concentration trials. Similarly, there was no significant difference in food consumption between the sulfoxaflor and control group (*p* = 0.1567) in the higher concentration sulfoxaflor trial (Figure 2B, Supplementary Table S2B).

### 3.2. Pesticide Impacts on Training Parameters

**Figure 3:**
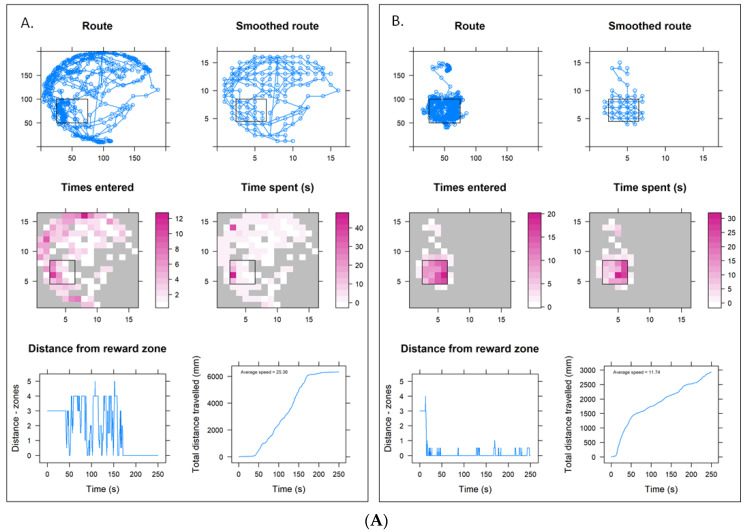

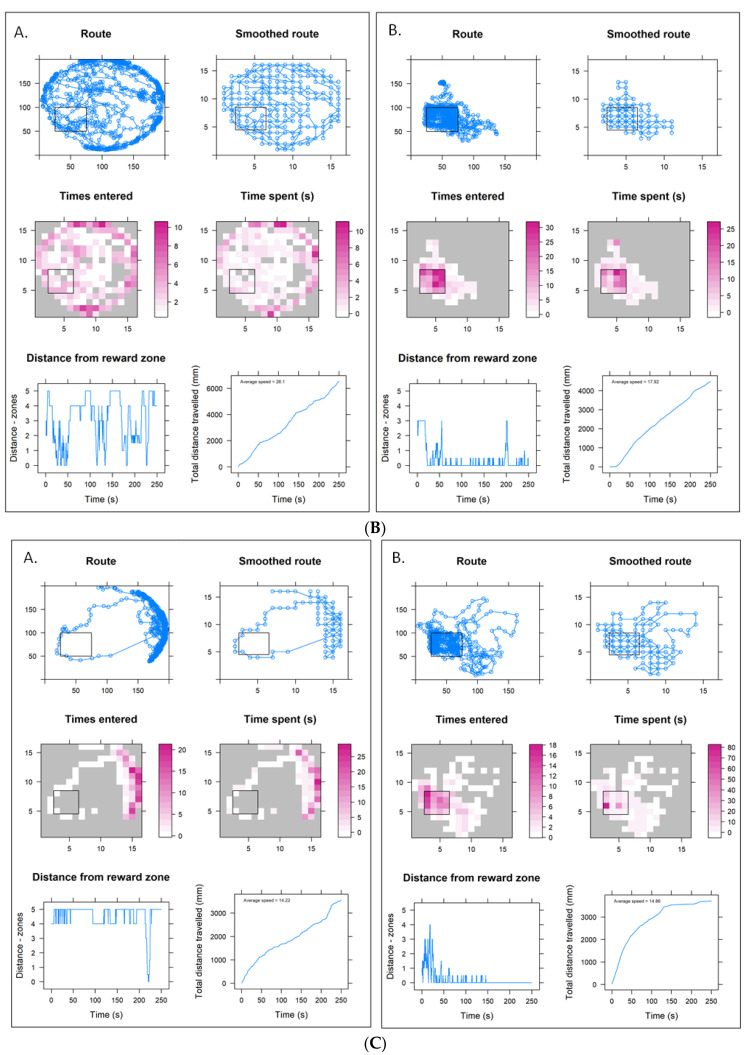

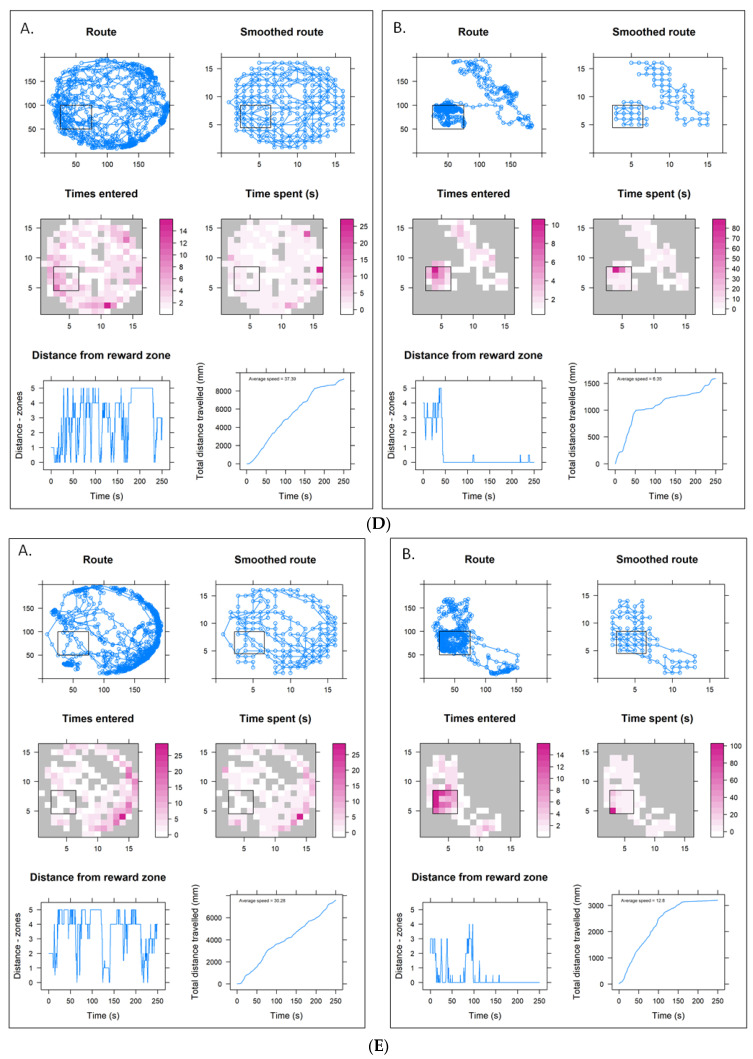
(**A**) Training parameters from a control bee (B63) pre- (panel A) and post- (panel B) training. Control condition identical between lower and higher concentration trials. In each case the black square outline indicates the cool tile reward zone location within the arena. (**B**) Training parameters from a lower concentration sulfoxaflor bee (B37) pre- (panel A) and post- (panel B) training. (**C**) Training parameters from a lower concentration thiacloprid bee (B56) pre- (panel A) and post- (panel B) training. (**D**) Training parameters from a lower concentration thiamethoxam bee (B100) pre- (panel A) and post- (panel B) training. (**E**) Training parameters from a higher concentration sulfoxaflor bee (B87) pre- (panel A) and post- (panel B) training.

Figure 3A–E give example training parameter trajectory graphs recorded pre- and post-training for one bee (of the nine bees tested) in each treatment group for both lower and higher concentration trials conducted in the thermo–visual arena.

### 3.3. Lower Concentration Insecticide Trials Data

**Figure 4:**
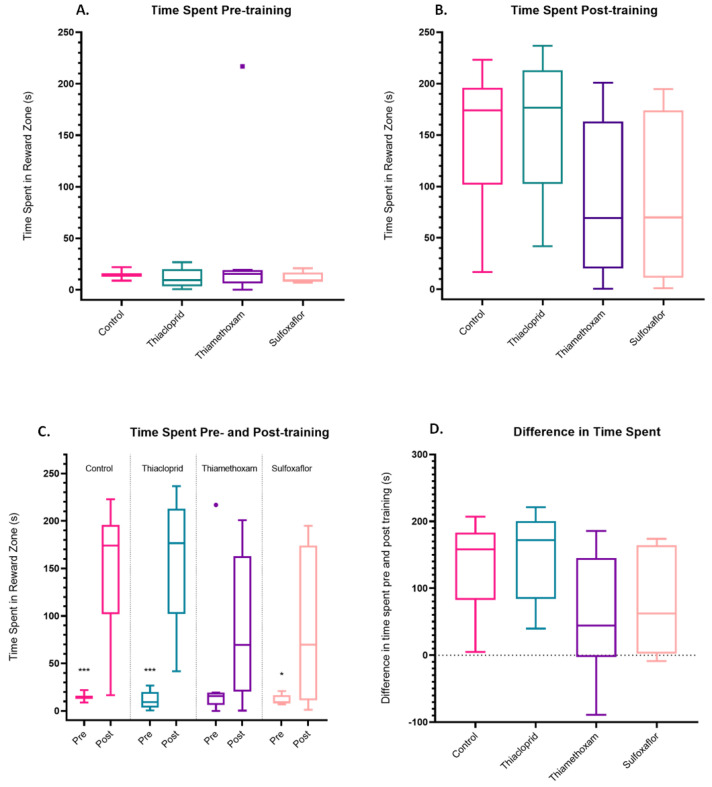
Time spent in the reward zone by bees in the lower concentration trials. (**A**) Time spent in the reward zone pre-training. *p* ≧ 0.99 for all comparisons (Kruskal-Wallis test with multiple comparisons). (**B**) Time spent in the reward zone post-training. *p* values range from 0.21 to 0.99 (one-way ANOVA with Tukey correction for multiple comparisons). (**C**) Time spent in the reward zone pre- versus post-training. Control (paired *t*-test, *p* = 0.0004 ***, *t* = 5.901, *df* = 8), thiacloprid (paired *t*-test, *p* = 0.0002 ***, *t* = 6.594, *df* = 8), sulfoxaflor (Wilcoxon matched-pairs signed rank test, *p* = 0.0195 *). Bees in the thiamethoxam treatment did not significantly increase the time they spent in the reward zone post training (versus pre-training) (Wilcoxon matched pairs signed rank test, *p* = 0.1289), but this is likely because one individual outlier (■) spent a large amount of time in the reward zone pre-training (216.8 s), as when this individual is removed the comparison becomes significant for all other bees in the thiamethoxam treatment (*p* = 0.0391 *). (**D**) Difference in time spent (post-training − pre-training value). A positive value indicates a bee improved, spending more time in the reward zone post-training whereas a negative value indicates the bee got worse at the task, decreasing the time spent in the reward zone post-training. Control *n* = 9, sulfoxaflor *n* = 9, thiacloprid *n* = 9; thiamethoxam *n* = 9.

The time spent in the ‘cool’ reward zone within the thermal–visual arena was analysed for each individual bee. Pre-training, there was no significant difference in the time spent in the reward zone for any of the treatments (Figure 4A). Post-training, there was no significant difference in the time spent in the reward zone by any of the treatments (Figure 4B). Pre- versus post-training paired *t*-tests or Wilcoxon matched-pairs signed rank tests (dependent on whether data were normal or non-normal) were also conducted to assess whether bees within each treatment had significantly increased their time spent in the reward zone post-training, compared to their own pre-training values. All treatments, apart from the thiamethoxam bees, spent significantly more time in the reward zone post-training compared to their pre-training values (Figure 4C). However, the thiamethoxam data have been skewed by one individual bee spending a large portion of time in the reward zone pre-training (Figure 4C). Difference in time spent pre- and post-training was also calculated for each bee (post-training minus pre-training value). There were no significant disparities between the differences in time spent in the reward zone pre- and post-training for any of the treatment groups. This supports the notion that aversive training is still highly effective across all treatment groups, regardless of insecticide exposure at the lower concentration values (Figure 4D).

**Figure 5:**
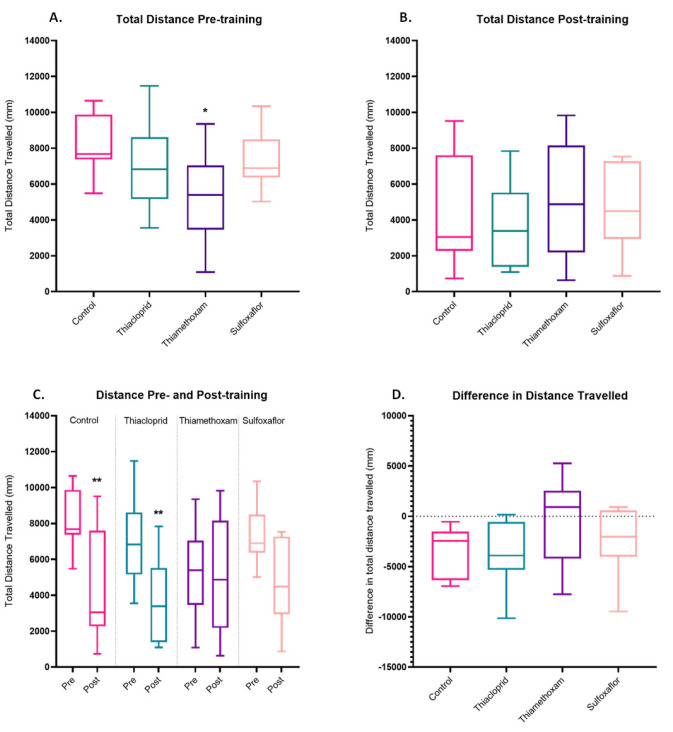
Distance travelled by bees in the lower concentration trials. (**A**) Total distance travelled pre-training. One-way ANOVA with Tukey correction for multiple comparisons, thiamethoxam *p* = 0.027 *, thiacloprid *p* = 0.66, sulfoxaflor *p* = 0.72. Thiacloprid vs sulfoxaflor *p* = 0.99, thiamethoxam vs. sulfoxaflor *p* = 0.24, thiamethoxam vs thiacloprid *p* = 0.28. (**B**) Total distance travelled post-training. One-way ANOVAs with Tukey correction for multiple comparisons, control vs. thiacloprid *p* = 0.86, control vs. thiamethoxam *p* = 0.99, control vs. sulfoxaflor *p* = 0.99, thiacloprid vs. thiamethoxam *p* = 0.67, thiacloprid vs. sulfoxaflor *p* = 0.75 and thiamethoxam vs. sulfoxaflor *p* = 0.99. (**C**) Total distance travelled pre- versus post-training. Paired *t*-tests, control *p* = 0.0022 **, thiacloprid *p* = 0.0092 **, sulfoxaflor *p* = 0.06, thiamethoxam *p* = 0.817. (**D**) Difference in distance travelled (post-training − pre-training value). A negative value is indicative that a bee travelled less distance post-training vs. pre-training. Control *n* = 9, thiacloprid *n* = 9, thiamethoxam *n* = 9, sulfoxaflor *n* = 9.

The total distance travelled within the arena by each individual bee was also assessed. Pre-training, thiamethoxam bees travelled significantly less distance than control bees. Neither the thiacloprid or sulfoxaflor groups travelled significantly different distances to the controls or each other and the thiamethoxam group was not significantly different from the sulfoxaflor or thiacloprid groups (Figure 5A). Post-training, there were no significant treatment comparisons, with none of the groups travelling significantly different distances to each other (Figure 5B). Pre- vs. post-training paired *t*-tests were conducted to assess whether bees within each treatment had significantly altered the distanced travelled within the post-training trial versus the pre-training trial. Control and thiacloprid bees significantly reduced the distance they travelled in the post-training trial versus the pre-training trial. However, the sulfoxaflor and thiamethoxam bees did not significantly alter the distance travelled post-training. However, it should be noted that the sulfoxaflor group (*p* = 0.06) was just 0.01 away from the significance threshold of 0.05, suggesting a general reduction in distanced travelled, although not significant. The thiamethoxam bees had a much higher significance level of *p* = 0.8, implying very little change between pre- and post-training (Figure 5C). The difference in distance travelled pre- and post-training was then calculated for each bee. There were no significant differences between the differences in distance travelled pre- and post-training for any of the treatment groups. However, as Table 1 shows, there is a large difference between the treatment means here, suggesting that thiamethoxam bees improved the least (the least negative value) between pre- and post-training trials (Figure 5D).

**Figure 6:**
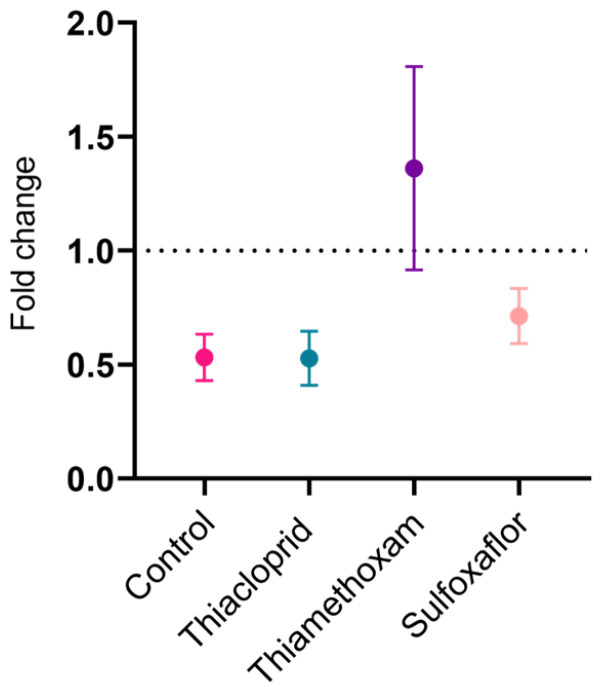
Bee speed fold change pre- versus post-training in the lower concentration trials (±SEM). Fold change calculated by dividing speed post-training by speed pre-training for individual bees. Control *n* = 9, thiacloprid *n* = 9, thiamethoxam *n* = 9, sulfoxaflor *n* = 9.

The average speed at which each individual bee travelled was also looked at in terms of fold change pre- versus post-training for each bee in each treatment. A fold change of 1 would indicate that a bee did not change its speed, a positive value that speed increased post-training and a negative change would indicate a bee decreased its speed post-training. There was a mean fold change in speed of 0.53 for the control group, 0.53 for thiacloprid, 1.36 for thiamethoxam and 0.71 for sulfoxaflor between pre- and post-training trials (Figure 6). This indicates that control and thiacloprid bees virtually halved their speed by the post-training trial. Sulfoxaflor bees also decreased their speed, whereas thiamethoxam bees, on average, increased their speed post-training.

### 3.4. Higher Concentration Sulfoxaflor Trials Data

**Figure 7:**
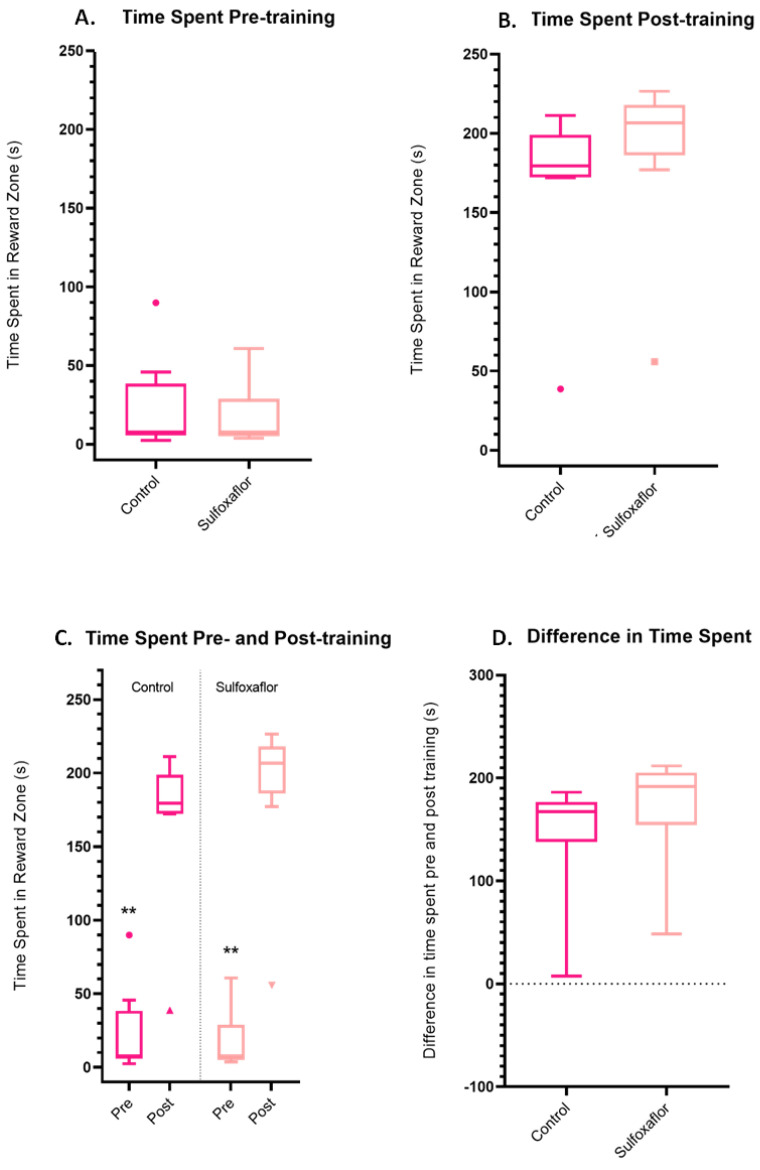
Time spent in the reward zone pre- and post-training by bees in the high-dose trials. (**A**) Time spent pre-training. *p* ≧ 0.99 for all comparisons, Kruskal-Wallis test. (**B**) Time spent post-training. Control vs. sulfoxaflor *p* ≧ 0.99. (**C**) Time spent pre- versus post-training. Control *p* = 0.004 **, sulfoxaflor *p* = 0.004 **. (**D**) Differences in time spent (post-training − pre-training value). Control vs. sulfoxaflor *p* ≧ 0.99. Control *n* = 9, sulfoxaflor *n* = 9. Coloured circles (•) and triangles (▲▼) represent individual bee outlier data.

For the higher concentration sulfoxaflor experiments, pre-training, there were no significant differences in the time spent in the reward zone by any treatments (Figure 7A). There were also no significant discrepancies between the differences in time spent in the reward zone post-training for the higher concentration sulfoxaflor treatment group (Figure 7B). Pre- versus post-training paired *t*-tests were also conducted to assess whether bees within each treatment (control vs sulfoxaflor) had significantly increased their time spent in the reward zone post-training, compared to their own pre-training values. Control bees spent significantly more time in the reward zone post-training compared to their pre-training values, as did sulfoxaflor bees (Figure 7C). There were no other significant discrepancies between the differences in time spent in the reward zone pre- and post-training for the control or sulfoxaflor treatment group (Figure 7D).

**Figure 8:**
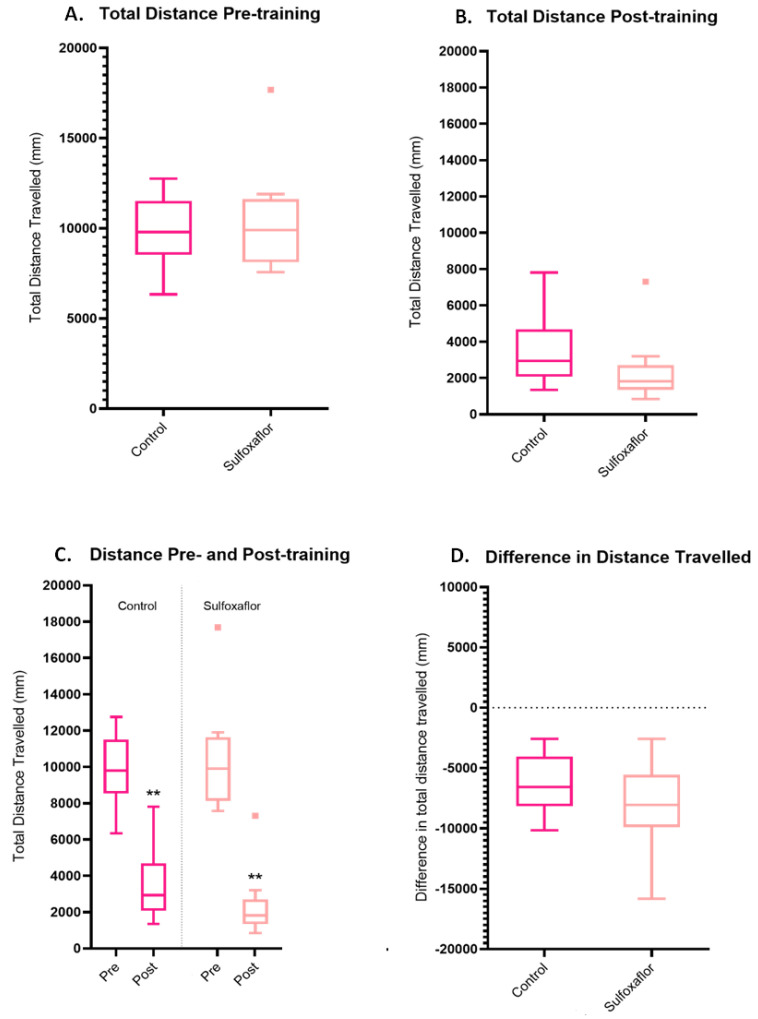
Total distance travelled pre- and post-training by bees in the high concentration trials. (**A**) Total distance travelled pre-training. *p* values > 0.7, Kruskal–Wallis test. (**B**) Total distance travelled post-training. Control vs. sulfoxaflor *p* = 0.93, Kruskal–Wallis test. (**C**) Total distance travelled pre- versus post-training. Control *p* = 0.0039 **, sulfoxaflor *p* = 0.0039 **. (**D**) Difference in distance travelled (post-training − pre-training value). Control *n* = 9, sulfoxaflor *n* = 9. Coloured squares (■) represent individual bee outlier data.

Pre-training, there were no significant differences in the distances travelled between control and sulfoxaflor-treated bees (Figure 8A). Post-training, there were no significant treatment comparisons, with neither the control nor sulfoxaflor-treated bees travelling significantly different distances to each other (Figure 8B). Pre- versus post-training paired *t*-tests were also conducted to assess whether bees within each treatment had significantly altered the distanced travelled within the post-training trial versus the pre-training trial. Control and sulfoxaflor bees all significantly reduced the distance they travelled in the post-training trial versus the pre-training trial (Figure 8C). There were no significant differences between the differences in distance travelled pre- and post-training for either of the treatment groups (one-way ANOVA) (Figure 8D).

**Figure 9:**
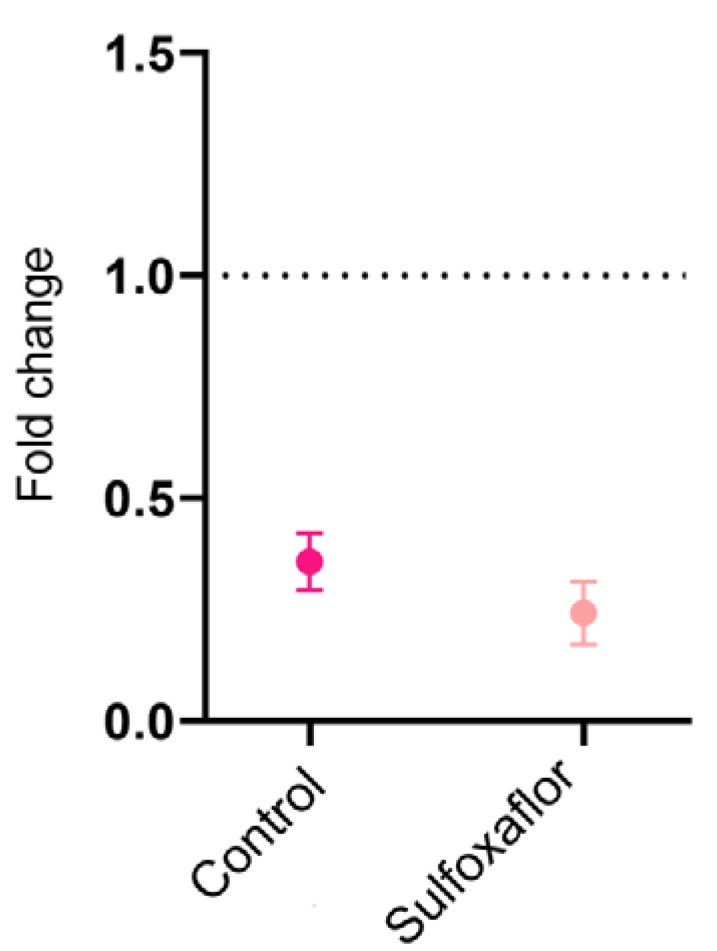
Bee speed fold change pre- versus post-training across treatments in the higher concentration trials (±SEM). Fold change calculated by dividing speed post-training by speed pre-training for individual bees. Control *n* = 9, thiacloprid *n* = 9, thiamethoxam *n* = 9, sulfoxaflor *n* = 9.

The average speed at which each individual bee travelled in the higher concentration sulfoxaflor trial was also determined. There was a mean fold change in speed of 0.36 for the control group and 0.24 for sulfoxaflor between pre- and post-training trials (Figure 9). This indicates that control and sulfoxaflor bees more than halved their speed by the post-training trial.

### 3.5. Speed–Curvature Power Law Analysis

**Figure 10:**
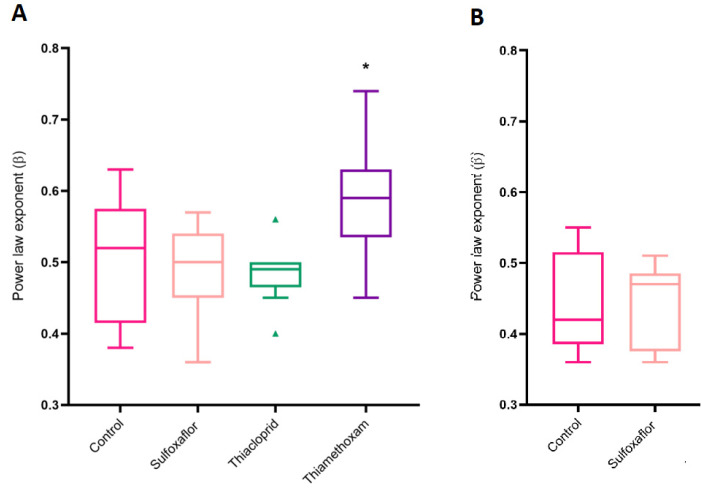
(**A**) β-exponent of bees in the lower concentration insecticide experiments post-training. Mean speed–curvature power law exponents for control 0.51 (range = 0.39:0.63), sulfoxaflor, 0.49 (range = 0.36:0.57), thiacloprid 0.49 (range = 0.4:0.56) and thiamethoxam 0.59 * (range = 0.45:0.74). Control *n* = 9, sulfoxaflor *n* = 9, thiacloprid *n* = 9 and thiamethoxam *n* = 9. Coloured triangles (▲) represent individual bee outlier data. (**B**) β-exponent of bees in the higher concentration insecticide experiments post-training. Mean speed–curvature power law exponents for control = 0.44 (range 0.36:0.55), sulfoxaflor = 0.44 (range 0.36:0.51). Control *n* = 9, sulfoxaflor *n* = 9.

In the lower insecticide concentration experiments, the mean speed–curvature power law exponents for the lower concentration treatments were 0.51 (control), 0.49 (sulfoxaflor), 0.49 (thiacloprid) and 0.59 (thiamethoxam). The thiamethoxam treatment group had the highest mean power law exponent and the largest deviation range of any of the groups, whereas there was no significant difference in the observed speed–curvature power law exponents between the sulfoxaflor and control treatments (Figure 10A). The mean power law exponents for the higher insecticide concentration treatments were 0.44 (control) and 0.44 (sulfoxaflor) (Figure 10B).

## 4. Discussion

Time spent in the reward zone within the thermal–visual arena was identified as a key indicator of bee training in James et al. [2] and there were no significant inter-treatment comparisons pre- or post-training found in the lower concentration trials (Figure 7), indicating that aversive training remains a highly effective paradigm in the pesticide trials conducted here. When we look at the difference (improvement) between pre- and post-training parameters for individual bees, which is arguably an even better indicator of training, we see that even bees in the higher concentration sulfoxaflor trials improved significantly in the time they spent in the reward zone post training (Figure 7), indicating that they remained highly capable of responding to the aversive training task. The insecticide-treated bees (bar one thiamethoxam bee outlier) clearly still had the ability to vastly improve their performance in the measured metric. An inability to respond to aversive conditioning, implied by a lack of improvement in the ‘time spent in the reward zone’ parameter, could potentially extrapolate to detrimental implications for bee learning in the field, for example, an inability to make aversive associations, but that is not something we see here.

We examined a further proxy of learning in the thermal–visual arena, the total distance travelled by bees within a trial. We would expect that as bees learn a reward location (in this instance the cool reward zone), their routes to and from this reward become optimised, resulting in minimising travelling distances [42,94,95,96]. In the lower concentration trials, as expected, the control and thiacloprid bees significantly reduce the distance they travel pre- versus post-training (Figure 5). The sulfoxaflor-treatment bees were also very close to the significance threshold at *p* = 0.06, indicating that, although not significant, these bees also reduced the distance they were travelling post-training. The thiamethoxam bees were the only group which did not significantly reduce the distance they travelled pre- versus post-training (Figure 5).

Developing an efficient route between destinations is a common occurrence for foraging bees in the wild, allowing them to minimise travelling costs between foraging locations and nest sites [42,94,96], and relies on spatial learning and memory [94,97,98]. The suggested inability of the thiamethoxam bees in these trials to develop a more efficient route by reducing the distance they travelled to and from the cool reward zone, and thus reduce the overall distance travelled in the post training trials, could have potentially concerning implications. Foraging bees which are unable to streamline their routes presumably have greater energy expenditures and feeding requirements than bees which can minimise route travel. Jacob et al. [99] found that stingless bees (*Tetregonisca angustula*) increased the distance they travelled by fivefold and Tosi et al. [9] noted that honeybees increased flight duration (+78%) and distance (+72%) (on a flight mill) in response to acute thiamethoxam exposure. However, different effects are seen under chronic exposure, with Tosi et al. [9] finding that honeybees significantly decreased their flight duration (−54%) and distance (−56%). We can equate the duration of the chronic exposure period in the Tosi et al. [9] study (1–2 days of continual exposure) to the pre-training trials of our study (after 3 days of exposure). Pre-training, in the lower concentration trials, we also see a reduction in distance travelled (walking not flight), with thiamethoxam bees travelling significantly less distance than control bees (*p* = 0.027 *). This finding, paired with the thiamethoxam bees’ inability to streamline their navigational routes (minimise distance travelled pre- versus post-training), suggests that chronic thiamethoxam exposure could have potential impacts on bumblebee foraging efficiency.

Previous studies in honeybees have observed hyperactivity in response to acute thiamethoxam exposure [9]. In the lower concentration pilot trial(s) conducted here, the control and thiacloprid bees almost halve their speed between pre- and post-training trials, and sulfoxaflor-treated bees also reduce their post-training speed by around 25% (Figure 6). However, the thiamethoxam bees increased their speed post-training by over 25%. These results support previous findings of hyper-activity induced by thiamethoxam exposure [99], suggesting they may be maintained in the longer term. In the higher concentration sulfoxaflor trials, we see a similar pattern to the lower dose trials, with control and sulfoxaflor bees more than halving their speed post-training compared to their pre-training values (Figure 9).

The insecticide concentrations selected for testing of each insecticide compound were, in the case of the lower concentration trials, based on literature reports of pollen and nectar detects, making these thiamethoxam sublethal effect findings concerning, but not unsurprising given existing literature [4,5,6,7,8,9,10,11,12,14]. However, no significant differences were noted for sulfoxaflor across any of the parameters we studied. This is promising for the future use of sulfoxaflor as a replacement compound to the neonicotinoids. Sulfoxaflor bees demonstrated marked improvement in the time spent, distance and speed parameters studied, demonstrating very similar patterns to control bees across all areas. This suggests that the sulfoxaflor bees were highly capable of completing the aversive conditioning task presented by the thermal–visual arena. This is somewhat supported by previous findings of no detrimental effects of sulfoxaflor on olfactory learning or memory [27]. Dietary exposure to sulfoxaflor in this study also did not alter feeding regimes. However, it should be noted that no reproductive effects were measured here, and it is these which have previously had detrimental effects reported [26].

A better understanding of the basal behavioural templates of bees has the potential to provide a critical tool to study fine-scale sublethal pesticide effects. Fractal analyses (such as power law analyses) have emerged as an important tool to distinguish between systems which are operating in a normal versus pathological state [73,100,101]. In wider biological systems, stress has been demonstrated to lead to a reduction in both temporal and structural complexity e.g., in heart rate fluctuations [101], lung geometry [102] and plant branching architecture [103]. Stressors such as disease have been demonstrated to cause variations from optimal behavioural templates in animals [73,74,75]. Similarly, stressors such as disease load and pesticide exposure are prevalent in agricultural landscapes, and yet, relatively little is known as to how they may impact animal movement patterns, particularly of less well studied pollinator species such as the bumblebees. Monitoring of pollinator health is vital to accurately assess the impacts of agricultural management practices on key ecosystem service providers. Being able to detect subtle sublethal pesticide effects could add power to the toxicological assessment tools currently available.

Here, we further studied the speed–curvature power law discovered in the walking trajectories of *B. terrestris* foragers [2], this time in foragers exposed to different sublethal insecticide regimes, to determine whether power laws have the potential to be used as diagnostic tools for the sublethal impact of pesticides on pollinators. We predicted that pesticide exposure may lead to changes in the movement patterns of *B. terrestris*, which would be reflected in a change in the power law relationships observed as a movement template in James et al. [2] and in the untreated control bees of this study. As predicted, we see a disruption to the power law exponent template under certain exposure regimes. All the bee trajectories analysed in these insecticide experiments adhere to the speed–curvature power law we previously discovered in the walking trajectories of untreated bees [2]. However, under certain insecticide exposure regimes we see a very different power law relationship. In the lower concentration experiments, the speed–curvature power law exponent for the thiamethoxam bees is significantly higher than the sulfoxaflor and the thiacloprid groups (Figure 10). By T10 the mean exponents for bees in the lower concentration treatments were control 0.51, sulfoxaflor 0.49 and thiacloprid 0.49, whereas the mean exponent for thiamethoxam bees was significantly higher at 0.59. It appears that the control, sulfoxaflor and thiacloprid groups in this T10 phase of the study are characterised by a speed–curvature power law relationship of approximately a half, whereas thiamethoxam bees’ speed–curvature relationship is characterised by an exponent closer to two thirds, demonstrating that a sublethal concentration of thiamethoxam led to a change in the underlying movement patterns of the bees.

These changes in the movement patterns of the thiamethoxam exposed bees are noteworthy, as we can demonstrate that the walking trajectories of treated bees have changed in subtle, yet detectable ways. These changes are consistent with wider conclusions, for example, Macintosh et al.’s findings that physiological stressors (e.g., parasitism) affect the locomotion behaviour of wild Japanese Macaques [73]. The lack of significant difference in power law exponents of the sulfoxaflor and thiacloprid bees (relative to controls) is largely reflective of the lack of sublethal effects reported above for these compounds. No sublethal effects were observed for thiacloprid across any of the assessed training parameters. However, sulfoxaflor bees (unlike thiacloprid and control bees) did not significantly reduce the distance they travelled (pre- versus post-training) in the lower concentration trials (but this was very close to the significance threshold and bees showed a general reduction in distance compared to thiamethoxam bees). Nonetheless, sulfoxaflor bees showed no sublethal effects in other observed behavioural parameters (speed travelled, or time spent in the reward zone) in either the lower or higher concentration trials and did decrease their distance travelled post-training in the higher concentration trials, suggesting that the level of sublethal effects observed is well matched by the power law parameter here. Nevertheless, as a tool, the power law exponent may miss non-movement based behavioural changes under pesticide exposure. The significant effects of thiamethoxam on bee movement patterns mirror the sublethal findings. In the trials thiamethoxam bees travelled significantly less distance in the pre-training trial (versus controls) and did not decrease their distance travelled post-training, potentially suggesting physical impairment preventing them from travelling as far in the initial trials and then a further inability to learn or streamline the route in later trials. It therefore appears that power laws can provide a robust estimate of the presence of underlying physiological or biomechanical disruption in response to sublethal pesticide exposure.

The T1 (trial 1) control bees assessed here were under the same training regime as the “aversive” bees analysed in James et al. [2], and therefore we can compare these two “control” treatments in T1 to check whether the power law exponent is consistent across experiments. There was no significant difference in the speed–curvature power law exponents of the T1 control bees from James et al. [2] or the T1 control bees from this study (ANOVA with multiple comparisons, *p* = 0.58). Therefore, we can see that the method used to analyse power law exponents used in James et al. [2] and here is a reliable and reproducible way to study *B. terrestris* trajectories. Hence, power law analyses have clear weight when it comes to assessing sublethal effects of pesticide exposure on bee movements. Furthermore, power law analyses could be used to determine sublethal pesticide levels at which exponents, and therefore movement relationships, are not disrupted, and optimal behavioural templates are maintained.

Movement analyses, such as those conducted here, are clearly effective in detecting subtle changes to bee movement patterns. These changes may otherwise have been overlooked under other assessment paradigms which do not pick up such fine-scale changes, and yet such changes could still have very real-world implications for foraging bees in the wild. Power law analyses may therefore be an effective way to assess the general state of pollinator health under sublethal pesticide exposure regimes. The use of novel ecotoxicology tools, such as the thermal visual arena [2], to study sub-lethal effects in wild bee species (e.g., *B. terrestris*), when paired with speed–curvature power law analyses, could provide much needed insight into the causation of observed sublethal effects. The fact that the speed–curvature power law appears to be inherently linked to biomechanical, and not neuronal processes, facilitates finer-scale dissection of the causes of observed behavioural deviations from the norm. The ability to screen compounds in this way could further facilitate the design of smarter, more specific pesticides, if early screening can be used to rule out (or in) specific sublethal mechanisms (e.g., biomechanical effects) at an early stage. The thermal–visual arena, used as an ecotoxicology screening tool, could thus aid in decision making of which chemistries may provide safer bee profiles. Equally, such a tool could be used at the other end of the ecotoxicology pipeline, to discern the causation of behavioural abnormalities observed in developing ecotoxicological field studies (e.g., the new OECD bee homing flight test [104,105], which determines behaviours, but not their potential causes). There is a clear, continuing need for further research into sublethal effects of neonicotinoids, as well as for newer replacement compounds, across a wide variety of bee species, in order that the evidence can be fully presented, and accurate risk assessments finalized.

It is vital that alternative replacements to the neonicotinoids are assessed in a timely manner, so that we are not playing catch-up with potentially devastating deleterious effects, as has been the case for several of the neonicotinoids. Compounds such as thiamethoxam, imidacloprid or clothianidin should be tested alongside neonicotinoids thought to be less harmful to bees (e.g., thiacloprid) and potential replacement compounds such as sulfoxaflor. Too often compounds are tested in isolation, making it difficult to determine whether current, or indeed newer, compounds in fact have fewer sublethal effects than the compounds that have gone before them. A continuing problem in the assessment of pesticide impacts on wild pollinators, such as bumblebees, is that research gaps (honeybees are still predominantly used as the model organism) preclude accurate risk assessment due to a lack of information. This has resulted in the publication of reports stating that it is unclear whether compounds have deleterious effects on wild pollinators [106]. It is therefore vital that these knowledge gaps are filled as quickly as possible, using sound and field realistic methodologies, so that accurate assessments of pesticide impacts can be collated. Quantifying pesticide impacts on non-target species is vitally important in being able to identify the potential wider-reaching ecological impacts of compounds [107,108]. Of particular interest in this study is whether realistic levels of pesticide exposures can illicit negative behavioural impacts on *B. terrestris* (a model wild pollinator species), which have the potential to have knock-on effects on the wider ecosystem.

It is apparent from the pilot experiments conducted here that disruptions to simple movement patterns (e.g., power laws) can be used to elucidate underlying stressors and potential sublethal effects in bees, but this tool could be far wider reaching. The power law approach has not yet been extended to further agricultural stressors or to other beneficial invertebrates. Power laws could be used in further pollinator assessments, for example, in examining the physiological or behavioural stresses of bee virus infections or varroa infestations, or of poor diet and nutritional stress. Equally, power law analyses could be used to assess other beneficials’ (e.g., pest predators and parasitoids) responses to pesticide exposure. Currently, power law analyses remain vastly underutilised, but have the potential to allow us to detect a range of subtle changes in our native pollinators and beneficials in response to chemical stressors, nutritional defects and disease.

## Figures and Tables

**Table 1:** Statistics of differences in distance travelled (mm) pre- vs. post-training in the lower concentration trials.

Treatment	Control	Thiacloprid	Thiamethoxam	Sulfoxaflor
Mean	−3741	−3648	−334.4	−2422
Std. Deviation	2539	3209	4204	3328
Std. Error of Mean	846.5	1070	1401	1109

## Data Availability

The data presented in this study are available on request from the corresponding author.

## Supplementary Materials

The following supporting information can be downloaded at: https://www.mdpi.com/article/10.3390/toxics11030279/s1, Figure S1. Schematic summary of the bee trials. Table S1B: Raw speed curvature power law exponents for individual bees in the lower concentration insecticide experiments (post-training), calculated from bee tracking data (Control; *n* = 9, Sulfoxaflor; *n* = 9, Thiacloprid; *n* = 9, Thiamethoxam; *n* = 9). Table S1B: Raw speed curvature power law exponents for individual bees in the high dose insecticide experiments (post training), calculated from bee tracking data. Control T1; *n* = 9, Control T10; *n* = 9, Sulfoxaflor; *n* = 9. Table S2A: Total food consumption per bee in the lower concentration treatments over the course of the full five days of the experiment. Table S2B: Total food consumption per bee in the higher concentration treatments over the course of the full five days of the experiment.
