# Supplementary material for: A Sublethal Concentration of Sulfoxaflor Has Minimal Impact on Buff-Tailed Bumblebee (Bombus terrestris) Locomotor Behaviour under Aversive Conditioning
Source: Toxics. 2023 Mar 18;11(3):279. doi: 10.3390/toxics11030279 (PMC10057571; doi:10.3390/toxics11030279)
Supplement: Supplementary file 1 [file toxics-11-00279-s001.zip › toxics-2264783-supplementary.pdf]

Supplementary Table S1A: Raw speed curvature power law exponents for individual bees in the low dose insecticide experiments (post-training), calculated from bee tracking data. (Control; *n* = 9, Sulfoxaflor; *n* = 9, Thiacloprid; *n* = 9, Thiamethoxam; *n* = 9).

Control	Sulfoxaflor	Thiacloprid	Thiamethoxam
0.58	0.57	0.56	0.58
0.57	0.48	0.49	0.74
0.52	0.55	0.4	0.62
0.5	0.5	0.45	0.57
0.38	0.53	0.49	0.64
0.43	0.36	0.5	0.45
0.63	0.5	0.5	0.5
0.4	0.42	0.48	0.61
0.56	0.52	0.5	0.59

Supplementary Table S1B: Raw speed curvature power law exponents for individual bees in the high dose insecticide experiments (post training), calculated from bee tracking data. Control T1; *n* = 9, Control T10; *n* = 9, Sulfoxaflor; *n*=9.

**Control** (T1)	**Control** (T10)	**Sulfoxaflor**
0.54	0.4	0.47
0.51	0.51	0.49
0.59	0.42	0.51
0.59	0.47	0.47
0.58	0.4	0.36
0.57	0.36	0.46
0.5	0.37	0.48
0.42	0.55	0.39
0.43	0.52	0.36

Supplementary Table S2A: Total food consumption per bee in the low dose treatments over the course of the full five days of the experiment. Evaporation is taken into account in the calculations. Insecticide dosage consumed per bee (ng) calculated.

**Evaporation feeders (g)**	**Control solution consumption (g)**	**Control - evaporation (g)**
0.0213	0.5292	0.506866667
0.0308	0.871	0.848666667
0.0149	1.2303	1.207966667
	2.3411	2.318766667
	0.8505	0.828166667
Average evaporation (g):	1.1087	1.086366667
0.022333333	1.3813	1.358966667
	1.6002	1.577866667
	1.4223	1.399966667
**Thiacloprid solution consumption (500 ppb) (g)**	**Thiacloprid - evaporation (g)**	**Thiacloprid consumed (ng)**
0.4285	0.406166667	156.2179487
0.7849	0.762566667	293.2948719
2.5935	2.571166667	988.9102565
2.0486	2.026266667	779.3333335
1.5811	1.558766667	599.5256412
0.1671	0.144766667	55.67948731
1.7003	1.677966667	645.371795
1.8261	1.803766667	693.7564104
0.6984	0.676066667	260.0256412
**Sulfoxaflor solution consumption (5ppb) (g)**	**Sulfoxaflor - evaporation (g)**	**Sulfoxaflor consumed (ng)**
1.8727	1.850366667	7.116794873
0.2587	0.236366667	0.909102565
0.3447	0.322366667	1.239871796
0.2441	0.221766667	0.852948719

0.9514	0.929066667	3.573333335
0.6664	0.644066667	2.477179488
1.6421	1.619766667	6.229871796
1.1803	1.157966667	4.45371795
1.5689	1.546566667	5.948333335
**Thiamethoxam solution consumption (10ppb) (g)**	**Thiamethoxam - evaporation (g)**	**Thiamethoxam consumed (ng)**
2.0728	2.050466667	15.77282052
1.1128	1.090466667	8.388205131
1.5311	1.508766667	11.60589744
1.0132	0.990866667	7.622051285
2.0031	1.980766667	15.23666667
0.4304	0.408066667	3.138974362
0.967	0.944666667	7.266666669
1.4318	1.409466667	10.84205128
1.2923	1.269966667	9.768974362

Supplementary Table S2B: Total food consumption per bee in the high dose treatments over the course of the full five days of the experiment

Control solution consumption (g)	Control - evaporation (g)	
1.5433	1.520966667	
2.4051	2.382766667	
1.6933	1.670966667	
2.0987	2.076366667	
2.3487	2.326366667	
1.2267	1.204366667	
1.4829	1.460566667	
1.6991	1.676766667	
1.4758	1.453466667	
**Sulfoxaflor solution (50ppb) (g)**	**Sulfoxaflor - evaporation (g)**	**Sulfoxaflor consumed (ng)**
1.6498	1.627466667	62.59487181
2.5661	2.543766667	97.8371795
1.0842	1.061866667	40.84102565
1.3657	1.343366667	51.66794873
0.4603	0.437966667	16.84487181
1.8664	1.844066667	70.92564104
0.7626	0.740266667	28.47179488
1.2966	1.274266667	49.01025642
0.8758	0.853466667	32.82564104
