# Supplementary figures and images for: A Sublethal Concentration of Sulfoxaflor Has Minimal Impact on Buff-Tailed Bumblebee (Bombus terrestris) Locomotor Behaviour under Aversive Conditioning
Source: Toxics. 2023 Mar 18;11(3):279. doi: 10.3390/toxics11030279 (PMC10057571; doi:10.3390/toxics11030279)

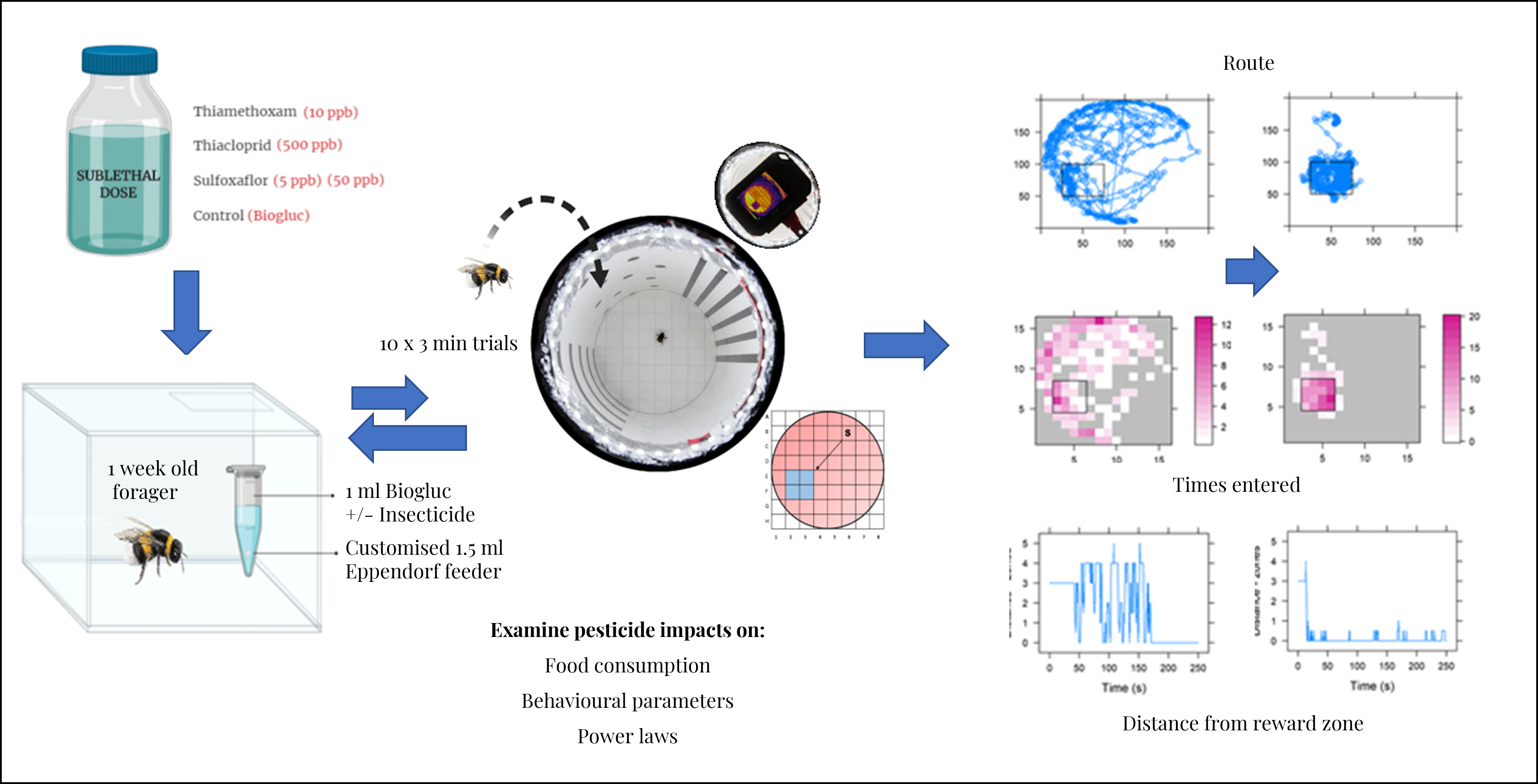

Supplement: Supplementary file 1 [file toxics-11-00279-s001.zip › Supplementary Figure S1.tif]
